# Alzheimer's Disease Mortality according to Socioeconomic Factors: Country Study

**DOI:** 10.1155/2018/8137464

**Published:** 2018-12-12

**Authors:** Peter Tóth, Beáta Gavurová, Miroslav Barták

**Affiliations:** ^1^Research and Innovation Centre Bioinformatics, TECHNICOM, Košice, Faculty of Economics, Technical University of Košice, Němcovej 32, 040 01 Košice, Slovakia; ^2^Department of Addictology, First Faculty of Medicine, Charles University and General University Hospital in Prague, Apolinářská 4, 128 00 Praha 2, Czech Republic

## Abstract

Aim of the paper is to quantify effects of socioeconomic factors on Alzheimer's Disease Mortality in the Slovak Republic. We applied potential gains in life expectancy (PGLE) method to measure the impact of elimination of Alzheimer's disease on life expectance in Slovak regions. PGLE is based on life table adjustment according to elimination of mortality caused by specific diagnosis. Our dataset consists of all deceased from Slovak Republic from 2001 to 2015. We analyse the impact of unemployment rate, GDP per capita, average wage, and education on life expectance in Slovak regions. To estimate that impact, ordinary least squares (OLS) is applied. According to our model, gross domestic product, average wage, and education influence mortality caused by Alzheimer's disease.

## 1. Introduction

Alzheimer's disease (AD) is degenerative brain disease and the most common cause of dementia. Dementia is a syndrome—a group of symptoms—which has several causes. AD is in the researches generally defined based on International Classification of Diseases and/or Statistical Manual of Mental Disorders (e.g., Prince et al. [1]). The discussion about how to measure AD is however much more complex as suggested, e.g., Alzheimer Europe discussion paper on ethical issues linked to the changing definitions/use of terms related to AD [2]. Characteristic signs of dementia are memory, language, problem solving, and other cognitive skills that affect a person's ability to perform day-to-day activities. Much of the AD increase will take in not very faraway future in low- and middle-income countries [3]. Furthermore, AD is an increasing issue in Central and Eastern European countries, including the Slovak Republic.

Qiu, Bäckman, and Winblad [4] estimated that 44 million people are living with dementia throughout the world and about 70% is caused by AD. AD has become global challenge and has large impact on both health and social systems (ibid). In 2015, dementia affected some 10.5 million citizens aged between 30 and 95+ years in Europe. This number is estimated to increase to 13.42 million people by 2030. According to Castro et al. [5], AD entails both direct and indirect economics cost. They suggest that indirect costs are more important in the early and community living AD patients and that direct costs are increasing with the disease progress.

Based on recent study of Reed et al. [6], total societal costs of caring for patients with AD vary across countries or regions of Europe with different health care systems. In their study, the mean 18-month societal costs per patient were in France €33,339, in Germany €38,197, and in the UK €37,899 (£32,501). According to Reed et al. [6], the caregiver time spent on basic and instrumental activities of daily living (ADL) contributed the most to societal costs (54% France, 64% Germany, and 65% UK). In 2016, Winblad et al. [7] found in their study of Spain, Sweden, the UK, and the USA that the costs are about EUR 14,500 annually in patients at home setting with higher level of autonomy but for residential care the cost may be higher than EUR 72,500. They also mention the geographical variations of AD as an important issue. Total estimated costs of dementia in 2015 were USD 818 billion. By 2018, dementia will become trillion-dollar disease, rising to USD 2 trillion by 2030.

According to the European Dementia Monitor 2017 [8], Finland, the UK (England), and the Netherlands were the countries which belong to the most dementia friendly policies in place. The countries were ranked using the categories of care availability, care affordability, treatment, clinical trials, research collaboration, dementia as a priority, dementia-friendliness, legal rights, international conventions, and last but not least care and employment rights. The Slovakia Republic placed at 19th and the Czech Republic 20th place out of 36 European countries. Based on the results, the study reports better outcomes in Central and Eastern Europe than in the Southern Europe. Based on data from this report, Slovakia Republic excelled in care and employment rights in comparison with other European counties (2nd place). The report also suggests that there are no fully funded care services in the Slovakia—the half of services require the copayments and the second half is self-funded. The availability of residential care and the day care are considered to be insufficient; however home care is considered to be sufficient. According to same source, Slovakia is ranking as the 11th country out of 36 European countries in participation in European dementia research collaborations and funding of pan-European dementia research initiatives.

Numerous studies, which are introduced below, address the impact of socioeconomic and genetic factors on Alzheimer's disease. Nicolia, Lucarelli, and Fuso [9] expanded these factors by the impact of various environmental factors (nutrients, pollutants, chemicals, physical activity, and lifestyle, physical and mental stress) to epigenetic markers. Moceri et al. [10] analysed the Alzheimer's disease risk associated with the father's occupation, the age of the parents, the size of the household, the number of siblings, and the birth. Kalaria et al. [11] selected as risk factors for dementia in the developing countries of Asia, Latin America, and Africa age (especially those over 65) and gender (women are more likely to develop dementia than men). Other risk factors include genetic and environmental factors (including early brain development, body growth, socioeconomic conditions, etc.), illiteracy, and educational levels. Ertekin et al. [12] present in their research study of the impact of risk factors on the prevalence of Alzheimer's disease in the Eastern Region of Turkey. The authors examined the relationship between the prevalence of Alzheimer's disease, socioeconomic characteristics, and comorbidities. The incidence of Alzheimer's disease in older age increases in both sexes but increases significantly in women. A statistically significant relationship was also found between the level of education and Alzheimer's disease. With the increasing level of education, the likelihood of Alzheimer's disease is decreasing. The occurrence of Alzheimer's disease was greater in widowed individuals than in married couples. The incidence of Alzheimer's disease was higher for manual workers than for others. Karp et al. [13] in their study examined whether the association between low levels of education and increased risk of Alzheimer's disease and dementia may be explained by the socioeconomic status based on the occupation. The results of their study show that low levels of education and low socioeconomic status expressions of low employment are individually linked to increased risk of Alzheimer's disease and dementia. Evans et al. [14] aimed at assessing the relationship between the three socioeconomic status indicators: education, occupational prestige, and income at risk of clinically diagnosed Alzheimer's disease. The results of their analyses show that socioeconomic status markers (education, prestige, and income) predicted the development of Alzheimer's disease (ranked among risk factors). In individual analyses, less years of formal school attendance, lower incomes, and lower job prestige indicated higher risk of Alzheimer's disease incidence. Wang et al. [15] compared the effects of cardiovascular, cancer, HIV, Alzheimer's disease, and kidney disease on the development of life expectancy by age, race, and gender. Alzheimer's and kidney disease are the only two diseases that have these days a growing mortality trend. From the study results, the degree of impact of each of the disease categories studied on the expected life span of the US population is determined by age, race, and gender.

The goal of the paper is the analysis of available data about mortality caused by Alzheimer's disease in Slovakia in order to quantify effects of socioeconomic indicators in life expectancy in Slovakia regions. The article fills the gap in knowledge, as similar analysis has not yet been performed in the Slovak environment; however there is growing evidence on the importance of analysed factors on AD related mortality in other countries.

## 2. Data and Methodology

### 2.1. Data

To reach the aim of the paper, we use three datasets. The first dataset is mortality in Slovak Republic provided by the National Health Information Centre (Národné Centrum Zdravotníckych Informácií) of the Slovak Republic. The second database is population data from the Statistical Office of the Slovak Republic (Štatistický úrad Slovenskej Republiky). The third source of our data is Eurostat, from which we use socioeconomic data.

Because of the data availability, we analyse time period from 2001 to 2015. Our mortality database consists of all deceased in Slovak Republic during analysed time period. In this paper, we study effects of socioeconomic indicators on the potential gain in life expectancy when deaths caused by Alzheimer's disease are eliminated. We select four socioeconomic indicators: Gross Domestic Product (GDP) per capita, average nominal monthly wage, unemployment rate, and education level. Education level in region is measured by the percentage of employees with tertiary education. It is assumed that this indicator is more appropriate than number of graduated in region because universities are distributed unevenly among Slovak regions and many students study and live in different regions as well as many of them study abroad. All these indicators measure the level of the region development. According to previous studies, education is factor that reduces risk of Alzheimer's disease.

There are other factors which can potentially affect the number of deaths caused by the AD. The health policy in the Slovak Republic is centralized; therefore we do not consider the impact of the health policy in the analysis of regional disparities. The study of the impact of the healthcare facilities is not relevant in case of the regional analysis, because of the fact that the Slovak Republic is small country; there are usually only several specialized healthcare facilities with nationwide scope.

### 2.2. Methodology

Methodology consists of several steps. The first step is to calculate abridged life tables for all causes of death and abridged life tables eliminating Alzheimer's disease. Then potential gain in life expectancy (PGLE) can be calculated. The last step is panel models estimation in order to quantify impact of selected socioeconomic factors on the PGLE.

Abridged life tables are computed for 5-year age groups except first age group, which is divided into age 0 and ages 1–4. Then, abridged life tables for Slovak regions consist of ages* x ϵ 0, 1, 5, 10,…, 90, 95*. Let _*n*_*q*_*x*_ be group probability of death for age* x* with length of the age group* n*, computed by (1), where _*n*_*m*_*x*_ is observed death rate expressed as _*n*_*m*_*x*_ = _*n*_*D*_*x*_/_*n*_*K*_*x*_, where _*n*_*D*_*x*_ is number of deaths and _*n*_*K*_*x*_ is mid-year population size, and _*n*_*a*_*x*_ denotes distribution of deaths. Typically, it is assumed that deaths are randomly distributed across the age interval; therefore _*n*_*a*_*x*_* = 2.5* [13].

(1) qxn=n.mxn1+mxnn−anx

Abridged life tables contain age (*x *to* x+n*), probability of death (_*n*_*q*_*x*_), number of surviving (*l*_*x*_), proportion of deaths in age group (_*n*_*d*_*x*_), person-years lived (_*n*_*L*_*x*_), person-years of remaining life in the cohort (*T*_*x*_), and life expectancy (*e*_*x*_) [14]. Number of surviving denotes the number of persons from the original population of 100,000 live births who survive to the beginning of each age interval and is computed by (2), for* l*_*0*_*=100,000* [14, 15].

(2) lx+n=lx.1−qxn

(3) dxn=lx−lx+n

Person-years lived explains the total time in years lived between two indicated birthdays and is expressed by the following equation:

(4) Lxn=lx+lx+n2

Then, person-years of remaining life (*T*_*x*_) can be calculated by the following equation:

(5) $$\begin{array}{c} T_{x}=L_{x}^{}+L_{x+1}+\ldots +L_{95} \end{array}$$

Life expectancy explains the expectation of life at a given age and is given by the following equation:

(6) $$\begin{array}{c} e_{x}=\frac{T_{x}}{l_{x}} \end{array}$$

It is necessary to calculate abridged life tables eliminating Alzheimer disease in order to quantify PGLE. Probability of death for age x with length of age group n eliminating cause of death i (_n_q_x-I_) is calculated by the following equation [16].

(7) qnx−i=1−pnxDnx−Dnxi/Dnx

Number of surviving (l_x-i_), proportion of deaths (_n_d_x-i_), person-years lived (_n_L_x-i_), person-years of remaining life in the cohort (T_x-i_), and life expectancy (e_x-i_) were calculated using formulas (2), (3), (4), (5), and (6), respectively. Only one expectation is age x = 95, when person-years lived (_n_L_95-i_) is given by (8) and person-years of remaining life in the cohort (T_95-i_) is given by (9) and (10).

(8) Ln95−i=l95−i+l95−i.q95−i2

(9) Tn95−i=α.Ln95−i

(10) α=e95.ln95Ln95

Potential gain in life expectancy is derived as difference between life expectancy eliminating cause i and life expectancy for all causes. Mathematically potential gain in life expectancy PGLE is explained by the following equation:

(11) $$\begin{array}{c} PGLE^{-i}=e_{x}^{-i}-e_{x} \end{array}$$

To analyse impact of socioeconomic factors on PGLE eliminating Alzheimer's disease, panel data analysis was applied. General linear panel model is defined by (12), where y_it_ is a dependent variable, *α*_it_ and *β*_it_ are regression coefficients, and u_it_ denotes a random disturbance term of mean 0. Index i = 1,…, n marks individual index, in our case regions in Slovak Republic and t = 1,…, T represents time index [17].

(12) $$\begin{array}{c} y_{it}=\alpha_{it}+\beta_{it}^{T}x_{it}+u_{it} \end{array}$$

Parameter homogeneity is often assumed that means *α*_it_ = *α* for all i, t and *β*_it_ = *β* for all i, t. This is standard linear model, so-called pooling model. If individual component in the general model is correlated with regressors x_it_, then *α*_it_ =*α*_i_ and it is called fixed effects model. If it is not, model is called random effects model. Therefore, we estimate three models: fixed effects model, random effects model, and pooling model. There are many tests how to identify which model is most appropriate. The suitability of the pooling model can be tested using Test of poolability. To decide between fixed effects model and random effects model, Hausman Test is commonly used which compares two sets of estimates [18]. Lagrange multiplier test of individual and time effects introduced by [18] is used to decide which effects are involved in database. Existence of serial correlation is tested by Breusch-Godfrey test [19]. In addition to serial correlation, cross-sectional dependence is tested using Pesaran CD test for cross-sectional dependence in panels [20].

Analysis and all outputs were realized in the R software environment [21].

## 3. Results and Discussion

The analysis is divided into two parts. Firstly, we describe the expected life length in Slovak regions for all cause of death as well as when Alzheimer's disease is eliminated. We analyse the potential gain in life expectancy (PGLE) in case of Alzheimer's disease elimination. The second part provides a study of the impact of socioeconomic factors on Alzheimer's disease as the cause of death.

### 3.1. Expected Life Length

**Figure 1:**
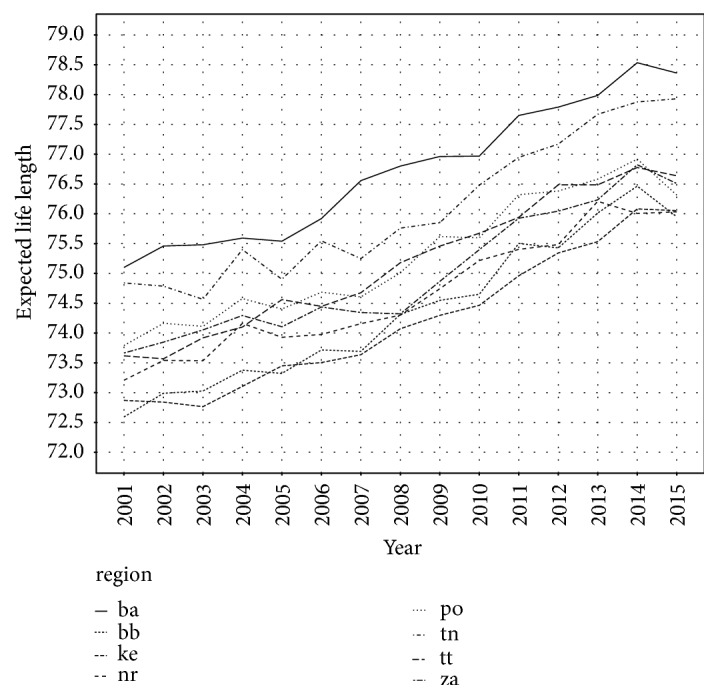
Expected life length in Slovak regions for all causes of death.

In general, there are significant regional disparities in the Slovak Republic. Western regions belong to the most developed regions with high GDP per capita and very low unemployment rate. On the other hand, eastern part of the Slovak Republic is typically less developed with high unemployment rate. That heterogeneity can be seen in case of expected life length, too. As Figure 1 shows, during all the studied period, Bratislava region (ba) has the highest expected life length. The second highest expected life length has Trenčín region (tn). Regions with the lowest expected life length are Kosice region (ke) and Banská Bystrica region (bb). Both these regions are less developed regions. It is important to emphasise that Prešov region (po), which belongs to the least developed regions, has the third highest expected life length, together with the Trnava region (tt). Life expectancy in Slovak regions at age 0 including all causes of death is presented in Table 1.

**Figure 2:**
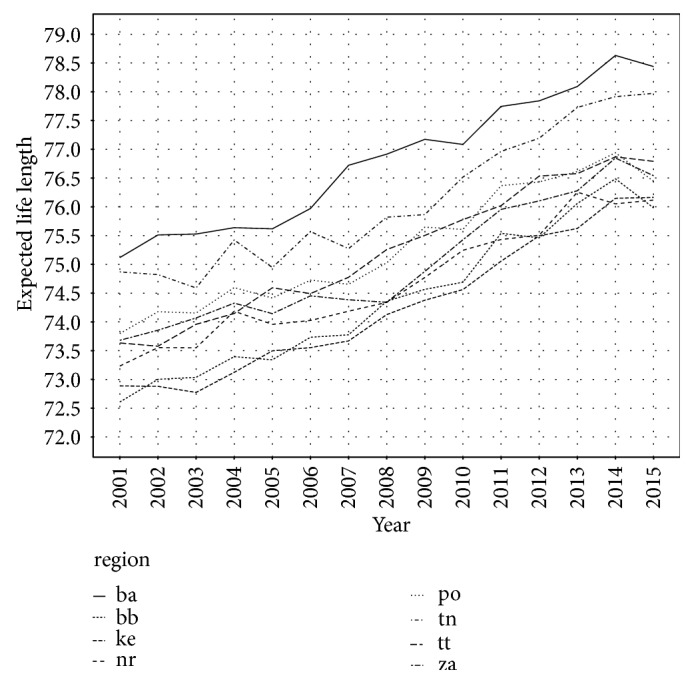
Expected life length in Slovak regions eliminating Alzheimer's disease.

Expected life length eliminating Alzheimer's disease in Slovak regions is depicted in Figure 2. Because of the small share of Alzheimer's disease on the causes of deaths, Figures 1 and 2 seem to be the same. But when we look more carefully, we can see several differences between them. Firstly, all values in Figure 2 are quite higher than in Figure 2. The second key aspect is that there are differences among regions, mainly in last years, when the number of deaths caused by Alzheimer's disease increased rapidly. In 2015, in Figure 1, there is Trnava region (tt) closer to the Žilina region (za) than in Figure 2. This means that Trnava region is affected by Alzheimer's disease more than Žilina region. It is because when eliminating Alzheimer's disease, expected life length increases more in case of Trnava region. In other words, Alzheimer's disease decreases real expected life length more in Trnava region as in the Žilina region. Life expectancy at age 0 by complete elimination of Alzheimer's disease for Slovak regions is shown in Table 2.

**Figure 3:**
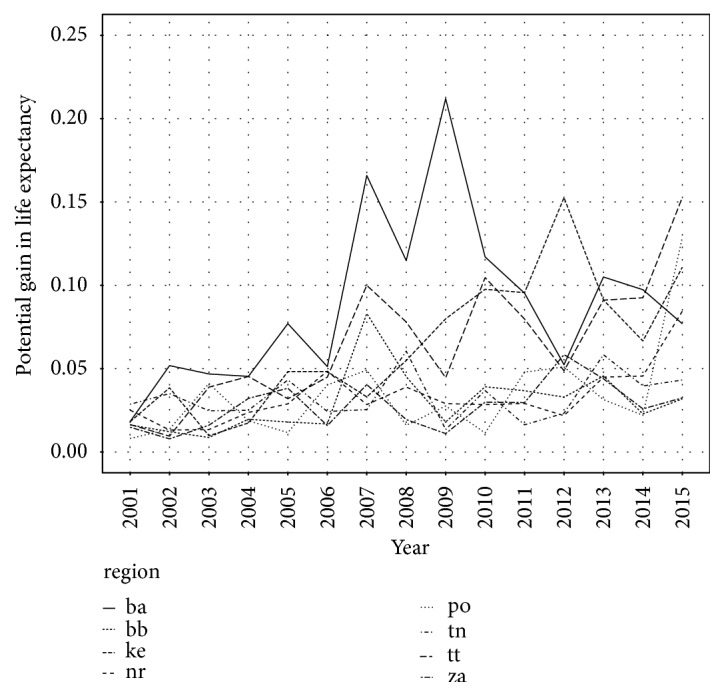
Potential gain in life expectancy by Alzheimer's disease elimination in Slovak regions.

Potential gain in life expectancy is calculated as difference between the expected life length for all causes of death and the expected life length excluding Alzheimer's disease.  Figure 3 shows development of the PGLE in Slovak regions during studied period. Higher PGLE explains that region suffers from Alzheimer's disease more. The most stricken regions are Bratislava region, Trnava region, and Kosice region. On the other hand, the least suffering regions are Banská Bystrica region, Trenčín region, Žilina region, and Nitra region. Prešov region increased in 2015 and is actually region with the second highest PGLE. In all eight Slovak regions PGLE has increased since 2001. The lowest PGLE is in Trenčín region, Žilina region, and Banská Bystrica region. Potential gain in life expectancy at age 0 by complete elimination of Alzheimer's disease for Slovak region is presented in Table 3.

### 3.2. Socioeconomic Indicators

In this section, we analyse the effect of socioeconomic factors on the potential gain in life expectancy. We study effects of gross domestic product per capita, average wage, unemployment rate, and share of the tertiary educated employees.

**Figure 4:**
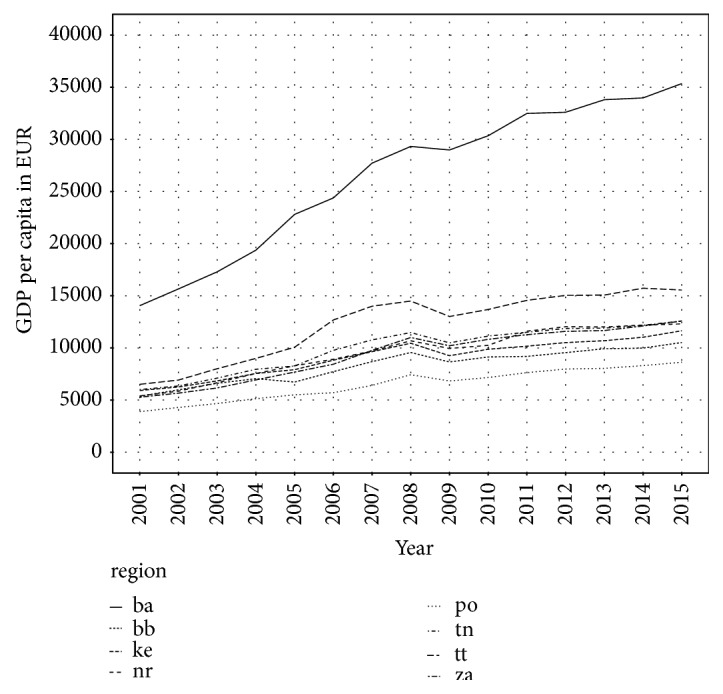
GDP per capita in Slovak regions.

As mentioned above, Slovak Republic is very heterogeneous country. There are significant regional disparities, which are nicely presented by regional GDP per capita, depicted in Figure 4. Bratislava region has the highest GDP per capita in each year. Negative aspect is that the difference between Bratislava region and other regions is still growing. In 2001, GDP per capita in Bratislava region was 15,000 EUR and in other regions was only 5,000 EUR. In 2015, it was 35,000 in Bratislava region and about 10,000 in other regions.

**Figure 5:**
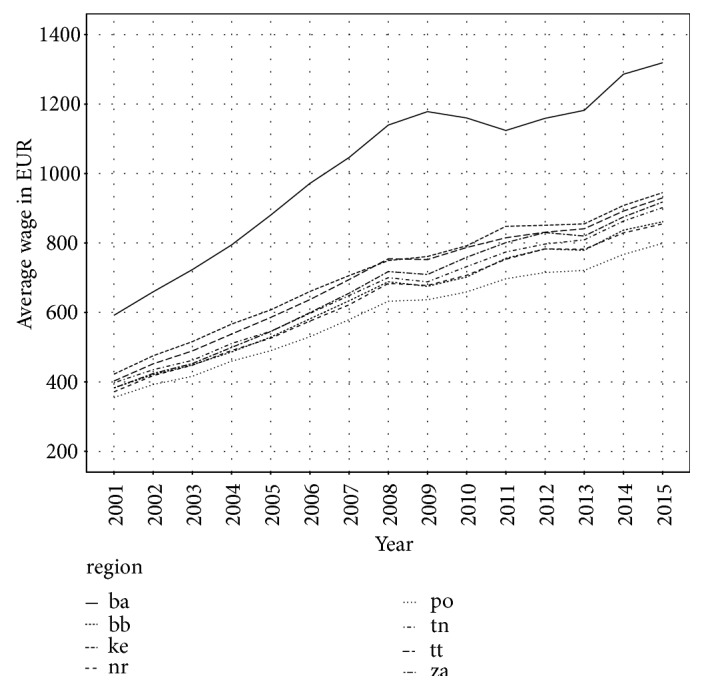
Average wage in Slovak regions.

The same situation is in average wage. The highest average wage is in Bratislava region. During studied period, it has increased from 600 EUR in 2001 to 1,100 EUR in 2015. The lowest average wage is in Prešov region in each year. It has grown from less than 400 EUR in 2001 to almost 800 EUR in 2015. The divergence between Bratislava region and group of other regions is significant. Average wage is shown in Figure 5, where we can see that average wage in Bratislava region reacted more sensitively on economic crisis in 2009 than wage in other regions.

**Figure 6:**
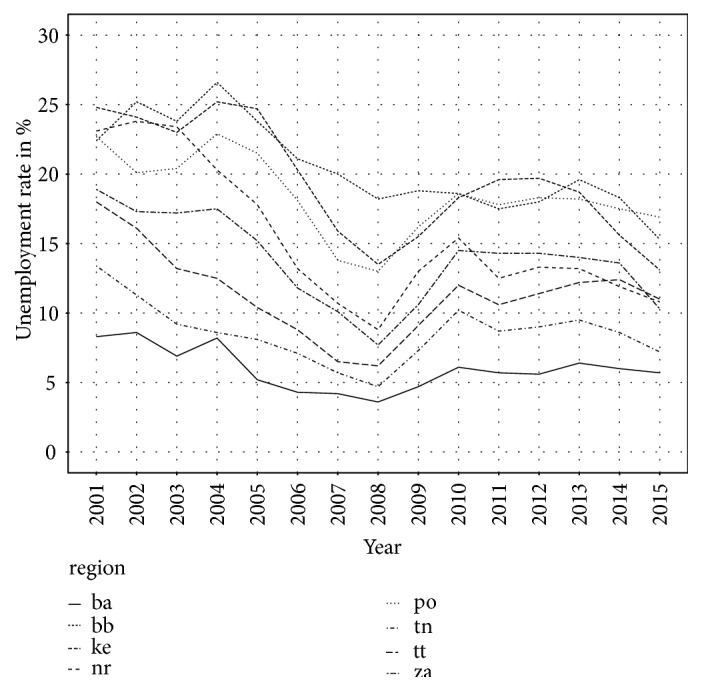
Unemployment rate in Slovak regions.

Unemployment rate was in Bratislava region the lowest in each year, which is shown in Figure 6. On the second place, there is Trenčín region and the third is Trnava region; all regions are located in the western part of the Slovak Republic. Among regions with the highest unemployment rate there are Prešov region, Banská Bystrica region, and Košice region, which are situated in the eastern part of the country and southern part in case of Banská Bystrica region. Here, we can see two positive aspects. First is that unemployment rate has declined in all regions and the second is that differences between the best and the worst region are decreasing.

**Figure 7:**
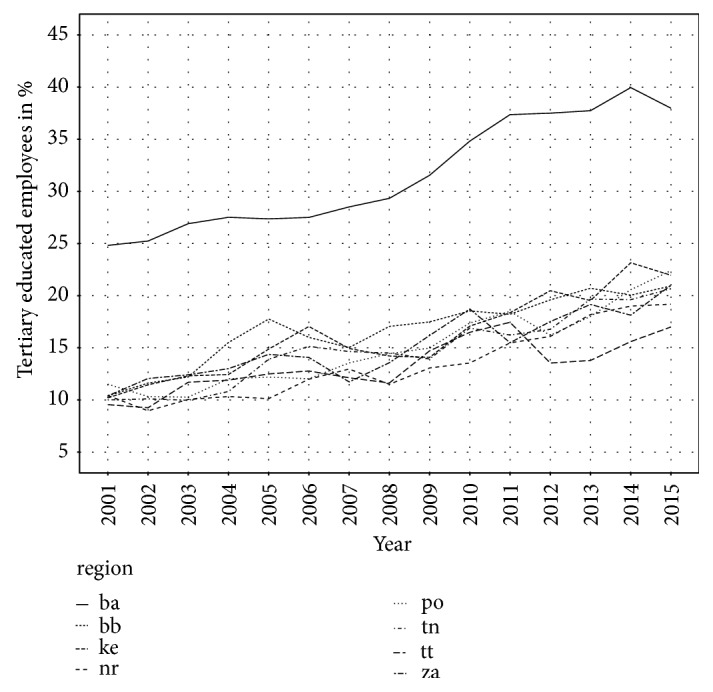
Percentage of tertiary educated employees in Slovak regions.

The last analysed socioeconomic factor is education, in our case the percentage of tertiary educated employees. As well as in the previous indicators, Bratislava region with the highest share of tertiary educated employees differs markedly from other regions. Percentage of tertiary educated employees has increased in all regions. The lowest value has Trnava region. Development of tertiary educated employees in Slovak regions is depicted in Figure 7.

### 3.3. Socioeconomic Effects on the PGLE

We applied panel data analysis to measure the impact of the selected socioeconomic indicators on the potential gain in life expectancy.

We estimated three linear panel models with PGLE as dependent variable and four independent variables: GDP, wage, unemployment, and education. First model is fixed effects model, second is random effects model, and the third is pooling model (3). According to poolability test, the most appropriate model is pooling model, which has also the highest R-Squared, too. This means that region does not affect the level of expected life length reduction caused by Alzheimer's disease. Estimates of all three models are presented in Table 4. We used logarithmic modification to ensure stationarity of variables.

Estimated regression coefficients from pooling model (3) show that statistically significant variables are GDP, wage and education. Estimated coefficient of variable GDP is positive, which means that the increase in change of the GDP in region causes the increase in change of the potential gain in life expectancy. This can be interpreted that number of deaths caused by Alzheimer's disease grows with increase in change of the GDP. The same situation is in case of the wage. Regression coefficient of the variable wage is positive; therefore the increase in change of the wage in the region causes increase in the change of the potential gain in life expectancy, which means more deaths caused by Alzheimer's disease. On the other side, estimated regression coefficient of the variable education has negative sign, so if we increase the change of the percentage of the tertiary educated employees, it leads to declination of the change of the potential gain in life expectancy, which means less deaths caused by Alzheimer's disease.

## 4. Discussion

Similar to other findings in literature, our study confirms that the number of deaths caused by Alzheimer's disease has increased rapidly. Due to the economic development and the improvement of social conditions over the last decades, life expectancy has grown, which might explain the increasing public health issue. In 2013, Abubakar et al. [22] found that Alzheimer's disease was one of the top 50 global causes of years of life lost, which was caused by the increase in the last years. According to Mathers and Loncar [23], Alzheimer's disease will be the seventh highest cause of death in high-income countries in the year 2030.

The main aim of the paper is the analysis of the relationship between Alzheimer's disease and socioeconomic factors as GDP, wage, and education. This is supported by previous studies which showed that these factors of socioeconomic status influence prevalence of the disease. Many large population-based studies have looked at the impact of socioeconomic status on the prevalence of dementia [13, 14, 24, 25]. The majority of these studies confirmed a clear relationship between the prevalence of dementia and low socioeconomic status. Fischer et al. [26] analysed patients with Alzheimer's disease and found out a strong association between age, individual annual income range, education, medical comorbidity, and a diagnosis of dementia, with increased age and medical comorbidity being the strongest predictors. The low socioeconomic status is often associated with insufficient access to the health care but this is not the case of Slovakia, where health care is accessible to all population. This might be a reason that GDP per capita and average wage increase Alzheimer's disease burden. Another reason of that fact can be that labour positions with high qualification and labour positions with low qualification do not differ in wage enough, especially in automotive industry. Goldbourt, Schnaider-Beeri, and Davidson [27] discovered from three- to sixfold increase in the prevalence of dementia among patients with low socioeconomic status versus those with high socioeconomic status. Lang et al. [28] found that living in an economically deprived locality results in a high prevalence of dementia, independent of other factors of socioeconomic status, such as annual income and education.

The relationship between education and Alzheimer's disease or dementia has been widely examined over the past decade [29, 30]. In our study, higher education level was linked with lower mortality rates. There are several studies with opposite findings. An inverse association between educational level and the risk of Alzheimer's disease or dementia has been reported in cross-sectional [31–34] and case-control studies [10, 35]. Several incidence studies [36] and pooled incidence data from Europe [37] also demonstrate an inverse association between education and Alzheimer's disease or dementia. In contrast, other incidence studies [38, 39] as well as autopsy-verified studies [40, 41] have failed to find any evidence for this reverse relationship. The data might have been influenced by neglection of the effects of potential confounders, such as cognitive functioning prior to dementia, vascular diseases, and socioeconomic status.

Katzman [29] proposed that education could postpone the clinical expression of dementia symptoms by increasing the neocortical synaptic density (the “brain reserve” hypothesis). Letenneur et al. [42] suggested that educational and occupational attainment provided a reserve against dementia, in that persons with higher educational and occupational attainment could cope with advanced pathologic changes of the disease more effectively by maintaining function longer (the “cognitive reserve” hypothesis). In addition, we assume that the observed inverse association may reflect an earlier detection of demented subjects with a low level of education irrespective of the underlying pathologic progress. Majority of brain reserve studies have used education as a brain-reserve measure [40–42]. In another study, Qiu et al. [4] suggest that a low level of education is associated with an increased risk of developing clinical Alzheimer's disease or dementia, particularly in women and in younger-old age. In addition, a low level of education was related to increased mortality of all causes, but not to mortality of subjects with Alzheimer's disease or dementia in the general population. A low educational level was significantly related to all-cause mortality, but not to the mortality of subjects with Alzheimer's disease or dementia. Alternatively, the observed association between educational level and incidence of Alzheimer's disease or dementia may partly reflect detection bias, by which subjects with a low level of education tend to be clinically diagnosed at an earlier point in time.

## 5. Conclusion

Aim of the paper was to analyse the impact of socioeconomic factors on the life expectancy in Slovak regions by complete elimination of Alzheimer's disease. It is calculated as difference between life expectancy eliminating Alzheimer's disease and life expectancy for all causes of deaths. We used potential gain in life expectancy method to quantify influence of Alzheimer's disease on the life expectancy. This method showed that there are significant differences among Slovak regions.

Potential gain in life expectancy was subsequently used as dependent variable in panel model analysis in order to identify the impact of socioeconomic factors on the life expectancy. We analysed effects of the gross domestic product per capita, average monthly wage, unemployment rate, and education level. According to our results, unemployment rate does not affect the mortality caused by Alzheimer's disease. On the other hand, gross domestic product, average wage, and education influence statistically significant mortality caused by Alzheimer's disease. Education has indirect effect on the mortality caused by Alzheimer's disease. This is the same result as results from previous studies. Increase in wage and gross domestic product leads to the increase in mortality caused by Alzheimer's disease. This is in opposite of other studies, because, according to these studies, higher level of socioeconomic status decreases prevalence to Alzheimer's disease.

## Figures and Tables

**Table 1:** Life expectancy (in years) at birth (age 0) for Slovak regions.

Year	Regions							
	ba	tt	tn	nr	za	bb	po	ke
2001	75.100	73.618	74.840	73.208	73.665	72.592	73.791	72.870

2002	75.459	73.568	74.787	73.540	73.847	72.990	74.163	72.841

2003	75.481	73.920	74.565	73.536	74.053	73.027	74.113	72.765

2004	75.592	74.101	75.400	74.161	74.294	73.377	74.578	73.106

2005	75.541	74.561	74.902	73.928	74.105	73.323	74.403	73.449

2006	75.920	74.447	75.546	73.976	74.437	73.716	74.686	73.505

2007	76.557	74.676	75.249	74.157	74.344	73.693	74.606	73.637

2008	76.801	75.179	75.760	74.300	74.322	74.313	75.011	74.072

2009	76.961	75.458	75.852	74.743	74.867	74.548	75.619	74.297

2010	76.968	75.673	76.481	75.216	75.398	74.651	75.596	74.465

2011	77.650	75.941	76.947	75.403	75.926	75.503	76.320	74.960

2012	77.790	76.486	77.170	75.484	76.044	75.428	76.382	75.343

2013	77.986	76.487	77.670	76.210	76.235	76.014	76.582	75.533

2014	78.534	76.776	77.876	76.007	76.823	76.464	76.918	76.082

2015	78.362	76.638	77.929	76.033	76.508	75.950	76.315	76.053

Note: ba: Bratislava region, tt: Trnava region, tn: Trenčín region, nr: Nitra region, bb: Banská Bystrica region, po: Prešov region, and ke: Košice region.

**Table 2:** Life expectancy (in years) at birth (age 0) by complete elimination of Alzheimer's disease for Slovak regions.

Year	Regions							
	ba	tt	tn	nr	za	bb	po	ke
2001	75.118	73.634	74.869	73.233	73.680	72.608	73.799	72.887

2002	75.511	73.578	74.822	73.554	73.855	73.002	74.177	72.880

2003	75.527	73.958	74.590	73.549	74.069	73.036	74.154	72.775

2004	75.638	74.146	75.425	74.185	74.326	73.397	74.597	73.123

2005	75.618	74.593	74.945	73.957	74.143	73.341	74.414	73.497

2006	75.971	74.492	75.570	74.025	74.452	73.733	74.726	73.552

2007	76.723	74.776	75.274	74.186	74.385	73.776	74.655	73.670

2008	76.915	75.257	75.820	74.339	74.341	74.359	75.027	74.127

2009	77.173	75.503	75.865	74.772	74.878	74.566	75.647	74.377

2010	77.085	75.778	76.518	75.245	75.429	74.690	75.608	74.562

2011	77.746	76.020	76.963	75.432	75.955	75.539	76.368	75.056

2012	77.842	76.535	77.193	75.506	76.103	75.461	76.433	75.496

2013	78.091	76.578	77.728	76.254	76.279	76.059	76.613	75.624

2014	78.632	76.869	77.915	76.052	76.849	76.487	76.940	76.149

2015	78.439	76.791	77.972	76.118	76.541	75.982	76.444	76.164

Note: ba: Bratislava region, tt: Trnava region, tn: Trenčín region, nr: Nitra region, bb: Banská Bystrica region, po: Prešov region, and ke: Košice region.

**Table 3:** Potential gains in life expectancy (in years) at birth (age 0) by complete elimination of Alzheimer's disease for Slovak regions.

Year	Regions							
	ba	tt	tn	nr	za	bb	po	ke
2001	0.018	0.016	0.029	0.025	0.015	0.016	0.008	0.018

2002	0.050	0.010	0.035	0.013	0.008	0.012	0.014	0.038

2003	0.047	0.039	0.025	0.013	0.016	0.008	0.041	0.010

2004	0.045	0.045	0.025	0.024	0.032	0.020	0.019	0.017

2005	0.077	0.032	0.043	0.029	0.038	0.018	0.012	0.048

2006	0.051	0.045	0.024	0.049	0.016	0.017	0.040	0.048

2007	0.166	0.100	0.025	0.028	0.040	0.083	0.049	0.033

2008	0.115	0.078	0.060	0.039	0.019	0.045	0.016	0.055

2009	0.212	0.045	0.014	0.029	0.011	0.018	0.028	0.080

2010	0.117	0.105	0.037	0.028	0.030	0.039	0.011	0.098

2011	0.095	0.080	0.016	0.029	0.030	0.037	0.048	0.096

2012	0.052	0.049	0.023	0.022	0.058	0.033	0.051	0.153

2013	0.105	0.091	0.058	0.045	0.044	0.046	0.031	0.091

2014	0.097	0.093	0.040	0.046	0.026	0.023	0.022	0.067

2015	0.077	0.153	0.043	0.085	0.032	0.032	0.129	0.111

Note: ba: Bratislava region, tt: Trnava region, tn: Trenčín region, nr: Nitra region, bb: Banská Bystrica region, po: Prešov region, and ke: Košice region.

**Table 4:** Estimated coefficients of linear panel models.

	Fixed effects model (1)	Random effects model (2)		Pooling model (3)	
	Estimate	Estimate		Estimate	
Intercept	-	-16.10361	*∗∗∗*	-19.69592	*∗∗∗*

Log(GDP)	0.00666	0.36724		0.61835	*∗*

Log(WAGE)	1.41820	1.38012	*∗∗∗*	1.45152	*∗∗∗*

Log(UNEM)	-0.19705	-0.01797		0.14204	

Log(EDU)	-0.00456	-0.25301		-0.47277	*∗*

R-Squared	0.35220	0.42397		0.49238	

Note: *∗∗∗*, *∗∗*, *∗* denote significance levels on 1, 5, and 10 percent, respectively. GDP represents gross domestic product per capita, WAGE is average wage, UNEM denotes unemployment rate, and EDU is percentage of tertiary educated employees. According to the poolability test for individual cross-sectional effects using fixed effects model, all coefficients, excluding intercepts, are equal for individual effects (F = 1.432) as well as for time effects (F = 0.770). F test confirmed an existence of individual effects (F = 2.916*∗∗∗*) and absence of time effects (F = 1,113). There is not present cross-sectional dependence according to the Pesaran CD test (Z = -0.351). Breusch-Godfrey test for serial correlation in panel models did not confirm an existence of the serial correlation (Chi-squared=21.769). Hausman test prefers application of the (Chi-squared=3.056) fixed effects model (1).

## Data Availability

The data comes from the database of the National Health Information Centre of the Slovak Republic, Statistical Office of the Slovak Republic, and Eurostat Directorate-General of the European Commission in Luxembourg.
